# Identification of Maize CC-Type Glutaredoxins That Are Associated with Response to Drought Stress

**DOI:** 10.3390/genes10080610

**Published:** 2019-08-12

**Authors:** Shuangcheng Ding, Fengyu He, Wenlin Tang, Hewei Du, Hongwei Wang

**Affiliations:** 1 Hubei Key Laboratory of Waterlogging Disaster and Agricultural Use of Wetland, Agricultural College, Yangtze University, Jingzhou 434000, China; 2 Engineering Research Center of Ecology and Agricultural Use of Wetland, Ministry of Education, Agricultural College, Yangtze University, Jingzhou 434000, China; 3 Hubei Collaborative Innovation Center for Grain Industry, Agricultural College, Yangtze University, Jingzhou 434000, China; 4 Department of Biotechnology, College of Life Science, Yangtze University, Jingzhou 434025, China

**Keywords:** phylogenetic analysis, CC-type GRX gene family, genetic variation, drought stress, maize, expression pattern

## Abstract

Global maize cultivation is often adversely affected by drought stress. The CC-type glutaredoxin (GRX) genes form a plant-specific subfamily that regulate plant growth and respond to environmental stresses. However, how maize CC-type GRX (*ZmGRXCC*) genes respond to drought stress remains unclear. We performed a TBLASTN search to identify *ZmGRXCCs* in the maize genome and verified the identified sequences using the NCBI conservative domain database (CDD). We further established a phylogenetic tree using Mega7 and surveyed known *cis*-elements in the promoters of *ZmGRXCCs* using the PlantCARE database. We found twenty-one *ZmGRXCCs* in the maize genome by a genome-wide investigation and compared their phylogenetic relationships with rice, maize, and *Arabidopsis*. The analysis of their redox active sites showed that most of the 21 ZmGRXCCs share similar structures with their homologs. We assessed their expression at young seedlings and adult leaves under drought stress and their expression profiles in 15 tissues, and found that they were differentially expressed, indicating that different *ZmGRXCC* genes have different functions. Notably, *ZmGRXCC14* is up-regulated at seedling, V12, V14, V16, and R1 stages. Importantly, significant associations between genetic variation in *ZmGRXCC14* and drought tolerance are found at the seedling stage. These results will help to advance the study of the function of *ZmGRXCCs* genes under drought stress and understand the mechanism of drought resistance in maize.

## 1. Introduction

Abiotic stresses, especially drought, seriously affect plant growth and reproduction. Drought stress has far-reaching impact and strong destructive power, mainly due to the increased levels of reactive oxygen species in plants, such as hydrogen peroxide and singlet oxygen [1]. These oxidative substances are highly active and toxic because they can lead to lipid peroxidation and damage of proteins, carbohydrates, and nucleic acids, thereby destroying biofilm, affecting cell structure and function, and ultimately resulting in oxidative stress [2,3]. Therefore, studying the mechanism of antioxidation employed by plants is critical to the improvement of drought tolerance in plants.

The main ways plants employ to alleviate oxidative stress include both enzymatic and non-enzymatic systems [4]. The enzymatic system in plant cells includes superoxide dismutase (SOD), catalase (CAT), ascorbic acid peroxidase (APX), and glutaredoxin (GRX) [4]. GRX is a small redox enzyme composed of approximately 100 amino acid residues. In the action to keep its substrates from being oxidized, GRX transfers electrons to its substrates and becomes oxidized; it returns to the reduced state by acquiring electrons from glutathione. GRXs are not only ubiquitous in plants but an essential component of the plant antioxidation system to keep proteins in their properly reduced state [5,6]. GRXs have been studied for their involvement in response to oxidative stress [6,7,8]. GRXs play a critical role in scavenging reactive oxygen species (ROS) to prevent damage [4] when plants suffer from drought stress accompanied by a massive accumulation of ROS [9]. GRXs were suggested to form one of the most important protein modification systems in plants [5]. Meanwhile, GRXs were found to participate in the oxidation-reduction homeostasis and ROS signal transduction in plant cells [10]. Besides, the protein substrates of GRXs are involved in all aspects of plant growth, including primary metabolism, iron/sulfur cluster formation, development, environmental adaptation, and stress response [6]. GRXs are; therefore, a class of global regulators.

Several studies have reported genome-wide identification of the *GRX* gene family [11,12,13]. Based on the sequences at their redox activity centers, *GRXs* are classified into six categories, namely CSY(C/S)-, CGF-, CC-type, and other groups with unknown functions [11,12,13]. CC-type *GRXs* are a plant-specific subgroup and are also known as the Roxy family in *Arabidopsis thaliana* [14,15]. In sequence composition, CC-type GRXs have their unique conservative active site motif. For example, they contain unique CC(M/L)(C/S) conservative active site motifs in *Arabidopsis* [16]; whereas in rice, the conservative active site motifs extends to C(C/G/F/Y/P)(M/L)(C/S/I/A) [5,11,15,17]. The first CC-type GRX was identified as a regulator of fetal development [14]. However, CC-type GRXs also participate in jasmonic acid (JA)/ethylene (ET)-mediated abiotic stress response through its interaction with TGA transcription factors (TFs) [11,18]. Additionally, CC-type *GRXS13* is crucial in limiting ROS production induced by basic and photooxidative stress [19]. Therefore, CC-type GRXs play a vital role in the crosstalk between ROS and ET. CC-type *GRXs* also participate in organ development and abiotic stress responses in other plants [11,20,21,22,23].

Drought stress greatly affects the productivity of crops, including maize, which is a primary staple food and provides industrial raw materials [24,25,26]. The completion of the maize genome sequence provides the opportunity to annotate and analyze the whole genome of maize [27]. In addition, previous evidence suggests that CC-type *GRXs* are candidate genes that regulate maize growth and response to environmental stresses [11,20,21,22,23]. With the goal to understand the role of *GRXs* in maize, here we comprehensively analyzed the phylogeny, gene structure, and chromosome location of the CC-type *GRX* family. We also analyzed the molecular evolution of the gene family, promoter analysis, gene expression patterns in various tissues, and response to drought stress. These results will provide a useful reference for future research on the function of CC-type *GRX* genes in maize.

## 2. Materials and Methods

### 2.1. Identification of CC-Type GRX Protein-Coding Genes in the Maize Genome

CC-, CG-, and CP-type GRXs of rice and *Arabidopsis* were retrieved from the report by Garg et al. (2010) [13]. The protein sequences of CC-, CG-, and CP-type GRXs were obtained from the Rice Genome Annotation Project database 6 for rice and from the TAIR database 9 for *Arabidopsis*. These sequences were used as queries to search the maize B73 genome (genome assembly: AGPv3) in Phytozome V12 by TBLASTN. All the retrieved maize sequences were further curated using the NCBI Conserved Domain Database (CDD) [28] to determine whether there was a PF00462 protein domain. Those genes encoding proteins containing a CC-type redox site motif were considered ZmGRXCC genes.

### 2.2. Gene Structure and Phylogenetic Relationships Analysis

Protein sequences of maize ZmGRXs were downloaded from Phytozome V12. (Berkeley, California, USA) To display the exon/intron structure, the coding sequence of each *ZmGRXCC* gene was aligned to its corresponding genome sequence, and then the schematic was generated using GSDS 2.0 [29]. To build a phylogenetic tree, rice, *Arabidopsis*, and maize GRX proteins were aligned by ClustalX2 [30] and manually adjusted. The phylogenetic tree was built using this alignment output based on a maximum likelihood method in MEGA7 [31]. The parameters used were as follows: pairwise deletion and bootstrap (1000 replicates).

### 2.3. Identification of Cis-Regulatory Elements in Promoters of ZmGRXCCs

To identify potential *cis*-regulatory elements in the promoter sequences of *ZmGRXCC* genes, the 1500 bp sequences of each *ZmGRXCC* gene upstream of the ATG start codon were selected from the maize genome as the promoter, and the promoter sequence was screened using PlantCARE [32]. The elements searched included Skn-1_motif (-GGGCGG-), CCGTCC-box (-CCGTCC-box-), CAT-box (-GCCACT-), and RY-element (-CATGCATG-) for development and metabolism; TGACG-motif (-TGACG-) and CGTCA-motif (-CGTCA-) for jasmonic acid responsiveness; GARE-motif (-AAACAGA- or -TCTGTTG-) for gibberellin responsiveness; TGA-element (-AACGAC-) for auxin responsiveness; ABRE (ABA-responsive element, -CACGTG- or -TACGTG-) and CE3 (coupling element 3, -CACGCG-) for ABA responsiveness; ERE (ethylene-responsive element, -ATTTCAAA-) for ethylene responsiveness; GC-motif (-CCCCCG-), LTR (-CCGAAA-) and box S (-AGCCACC-) for stress responsiveness; and MBS (MYB-binding site, -CAACTG- or -TAACTG-), DRE (dehydration-responsive element, -GCCGAC- or -ACCGAC-), T/G Box (-CACGTT-), EE (evening element, -AATATC-), MYCR (MYC-binding site, -CACATG-), and NACR (binding site of drought-inducible NAC TFs, -CACGCA-) for drought/dehydration responsiveness [33,34,35,36,37].

### 2.4. Expression Profile Analysis

#### 2.4.1. Plant Treatments

Seedling and adult leaves of maize inbred line B73 were subjected to drought. Seedling growth conditions and drought stress treatments of B73 were conducted according to the protocol of Wang et al. [38]. Briefly, hydroponic-cultured seedlings at the three-leaf stage were placed on a plate and subjected to dehydration (40%–60% relative humidity and 28 °C). To study the effect of drought stress on adult leaves of B73, water was intercepted after the eight-leaf stage (V8), and plants with sufficient water (40% of soil moisture) were used as control. Three replicates of the middle section of the flag leaf were collected from both drought-stressed and well-watered plants (control) at the twelve-leaf stage (V12), the fourteen-leaf stage (V14), the sixteen-leaf stage (V16), and the silking stage (R1). Leaf samples of seedlings or flag leaves of at least three replicates were frozen in liquid nitrogen and stored at −80 °C until RNA isolation.

#### 2.4.2. RNA Isolation and Real-Time PCR Analysis

Total RNA was isolated from leaf samples from seedlings and adult leaves using the TriZol Reagent (Invitrogen, Carlsbad, California, USA) according to the product manuals. The relative expression of *ZmGRXCCs* was measured using quantitative real time-PCR (RT-qPCR) in 96-well plates using the ABI7500 Real-Time PCR Systems (Applied Biosystems, Foster, California, USA). The PCR reaction system consists of 1 μg cDNA, 200 nM primers, and 5 μL SYBR Premix Ex Taq II (Takara, Dalian, China), and the reaction volume is 10 μL. The PCR reaction was conducted with the following conditions: 10 min at 94 °C, 40 cycles of 15 s at 94 °C, and 30 s at 60 °C. The expression level of the *ZmUbi-2* gene (UniProtKB/TrEMBL; ACC: Q42415) was used as the internal control. For quantification, the 2^−ΔCT^ formula was used, and variations (standard errors) were calculated from three technical replicates for each of the three biological replicates. Primers for qRT-PCR are listed in Table S2.

### 2.5. Association Analysis

Association analysis for *ZmGRXCCs* was conducted by using a previously reported mapping population [38]. The mapping population, containing 367 maize inbred lines and corresponding survival rate under drought, contains 556,000 single nucleotide polymorphism (SNP) markers, with the minor allele frequency (MAF) ≥ 0.05. The identified *ZmGRXCCs* harbored 159 SNPs in the coding region and both the 5′-, and 3′-untranslated region (UTR). Three statistical models including the general linear model (GLM) model, adjusting the first two principal components (PC2), and the mixed linear model (MLM) model (incorporating PC2 and a kinship matrix) were selected to identify the SNPs significantly associated with drought tolerance by using the TASSEL4.0 program [39,40].

## 3. Results

### 3.1. Forty-Five Maize GRXs, Including Twenty-One CC-Type GRX (ZmGRXCC) Genes, Were Identified

**Figure 1:**
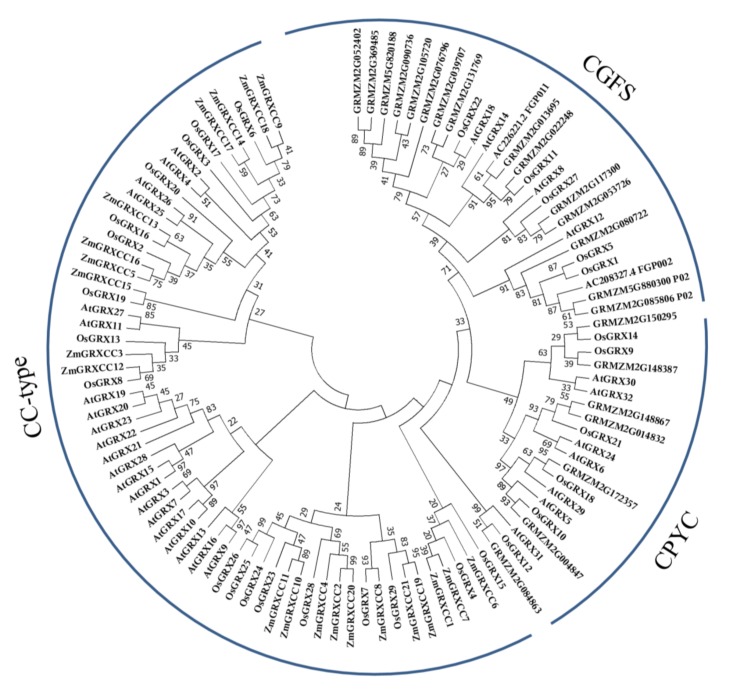
Phylogenetic tree of glutaredoxins (GRXs) from maize, rice, and *Arabidopsis*. Members of GRXs were classified by their redox activity site. The names used for GRX genes in rice and *Arabidopsis* followed the report of Garg et al. [13].

We systematically surveyed the maize genome to identify putative maize *GRX* genes. To further assess the phylogenetic relationship between GRX, we constructed a phylogenetic tree of maize, rice, and *Arabidopsis thaliana* GRXs (Figure 1, Table S1). As shown in Figure 1, a total of 106 GRX genes were divided into three types: CC, CP, and CG (Figure 1). We found that the CC-type subgroup had the most members among all the subgroups in maize. All *Arabidopsis* and rice GRX genes fell in the same class or clade as previously reported, in agreement with previous work [11,12]. Interestingly, the *Arabidopsis* GRXs were isolated from the two monocotyledons and clustered into a single branch in Figure 1.

**Figure 2:**
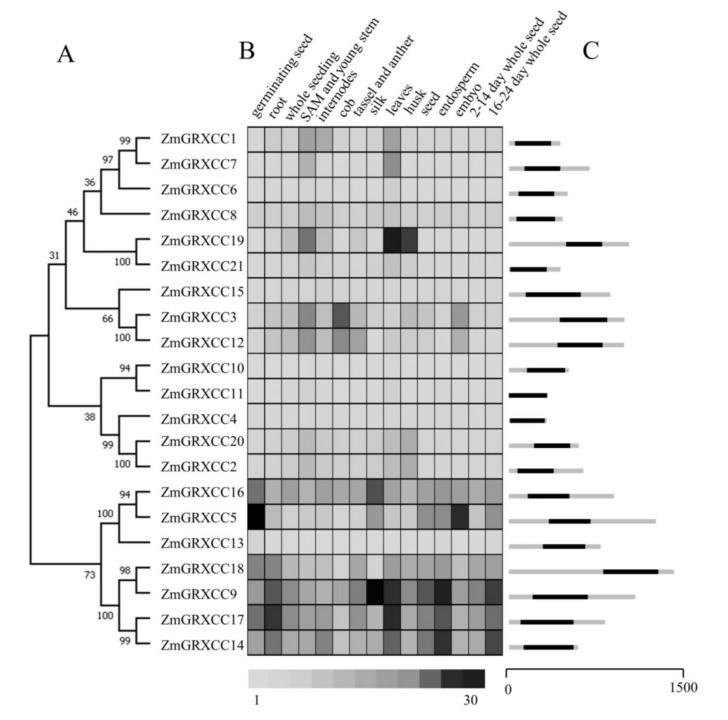
Predicted ZmGRXCC protein phylogeny, expression profiles, and gene structures. (**A**) Neighbor-joining (NJ) phylogeny of maize ZmGRXCC proteins. (**B**) Expression profile of maize *ZmGRXCC* genes. Normalized gene expression values [41] are shown in different colors that represent the relative levels of expression indicated on the scale bar. (**C**) Position of exons, introns, and untranslated region (UTR) in the *ZmGRXCC* genes. Introns were indicated by black boxes, UTR by gray box.

Among them, 21 contain the CC-type redox site and are thus ZmGRXCCs (Figure 1, Table 1). The physical location of each *ZmGRXCC* in the maize genome was determined based on the physical coordination provided by Phytozome (https://phytozome.jgi.doe.gov/pz/portal.html). Besides, the gene symbols are named according to their chromosomal locations (Figure 2, Table 1). The results showed that these 21 *ZmGRXCC* genes are unevenly distributed without clustering in the ten maize chromosomes except chromosome 9. Chromosome 3 possesses five *ZmGRXCC* genes, accounting for the largest number of *ZmGRXCCs* on a single chromosome. Chromosomes 8 and 10 each contain three genes, while chromosomes 5 and 7 each contain one *ZmGRXCC*.

### 3.2. Phylogenetic Relationship Analysis Showed High Conservation in ZmGRXCC Genes

We further studied the phylogenetic relationship, expression profile, and gene structure of the identified *ZmGRXCCs* (Figure 2). Tissue-specific expression profiles [39] based on the transcriptomic data of maize B73 were also used to reveal the roles of *ZmGRXCC* genes. An expression heatmap based on the normalized gene expression value was constructed for the 21 *ZmGRXCC* in different tissues, as well as developmental stages (Figure 2B). As shown in Figure 2B, the expression pattern of *ZmGRXCC* genes varied greatly. *ZmGRXCC5*, *ZmGRXCC9*, *ZmGRXCC13*, *ZmGRXCC14*, *ZmGRXCC16*, *ZmGRXCC17,* and *ZmGRXCC18* were expressed higher than other *ZmGRXCCs*. Interestingly, there were similarities between protein clustering and expression patterns. Proteins clustered together have similar expression patterns. Additionally, there is only one exon in each *ZmGRXCC* gene (Figure 2C), consistent with previous reports [12].

### 3.3. ZmGRXCC Proteins Were Highly Conserved Containing an Active Site and a C-Terminal ALWL Sequence

**Figure 3:**
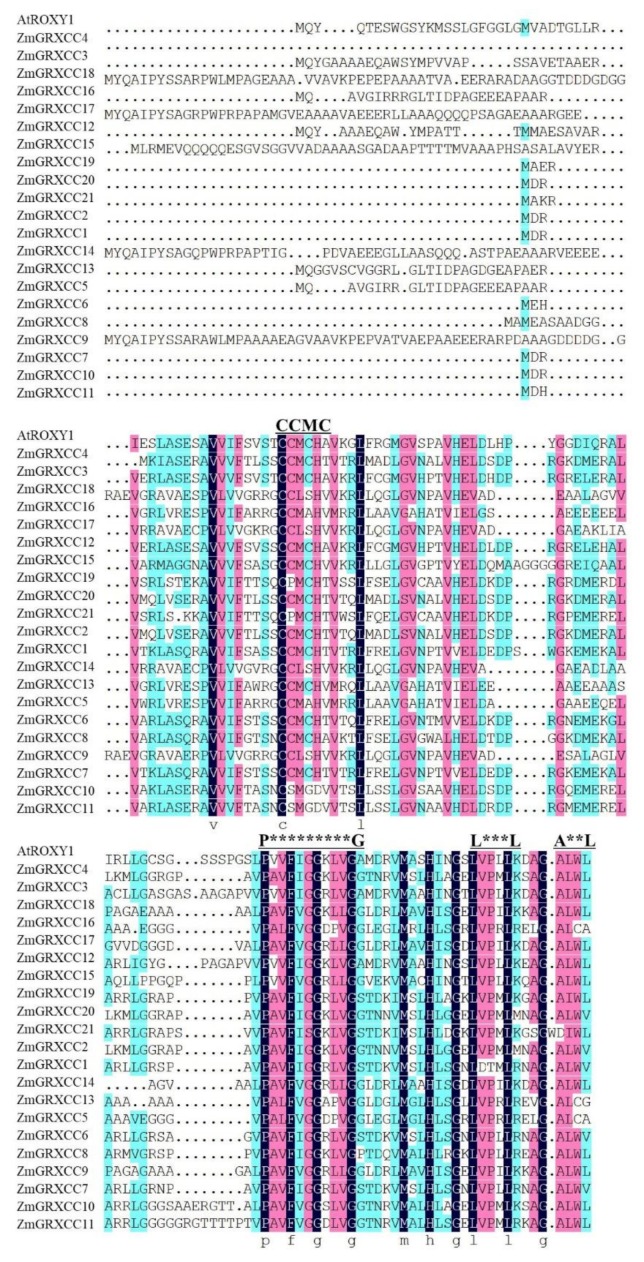
Alignment of ZmGRXCC protein sequences. Black boxes indicate identified conserved positions. The letters above the sequence indicate motif names.

Using the protein sequence of GRXs from rice and *Arabidopsis*, we found 21 ZmGRXCCs that contain a conserved redox activity site in the maize B73 genome (genomic version: AGPV3.0). To analyze the conserved motifs of identified ZmGRXCCs, we performed multiple sequence alignments of ZmGRXCCs and AtROXY1 (Figure 3). It was found that 11 of the 21 ZmGRXCCs shared an ALWL motif at the C terminus and was further extended to A(D)L(I)W(C)L(A/G/V) (Figure 3, Table 1). ZmGRXCCs also shared a distinctive CC(S/P)M(C/S) redox site (Figure 3, Table 1). The fact that the redox motif of ZmGRXCCs shares high sequence similarity with AtROXY1 demonstrates that CC-type GRXs undergo a relatively conserved evolutionary history (Figure 3, Table 1).

### 3.4. Promoter Analysis of the ZmGRXCC Gene Family

**Figure 4:**
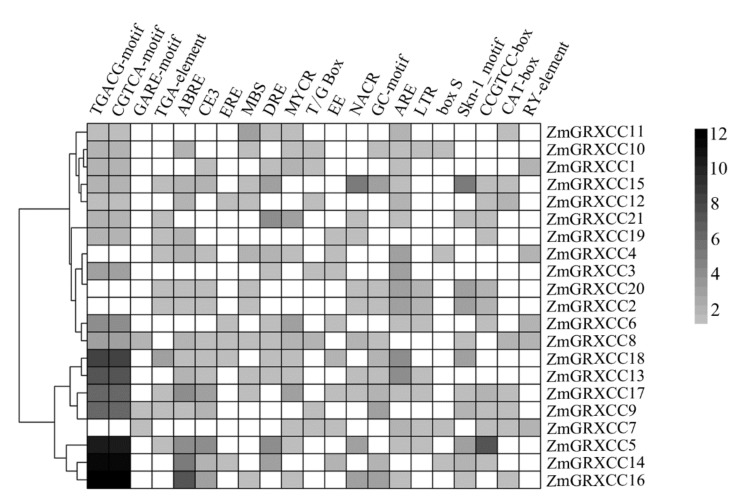
*Cis*-regulatory elements identified in the *ZmGRXCC* promoters. The 1.5 kb regions upstream of the translation start codons of the *ZmGRXCC* genes were used to search for putative *cis*-regulatory elements using the plant *cis*-acting regulatory DNA elements. The chart was drawn based on the presence and number of *cis*-regulatory elements that are responsive to specific elicitors or conditions. The color scale ranged from gray, representing minimum number of insertion, and finally to black, representing maximum number of insertion. The white color shows missing data.

We identified a total of twenty-one major regulatory elements in the promoters of *ZmGRXCC* genes; these elements are associated with phytohormones, growth, development, and abiotic stress (Figure 4). As shown in Figure 4, the genes belonging to the same subfamily have a diverse profile of *cis*-elements enriched in the promoters, suggesting a possible role for promoter sequences in functional diversification of the *ZmGRXCC* genes in the same subfamily. The presence of several major hormone-responsive elements, including the jasmonic acid-responsive element (TGACG-motif and CGTCA-motif), gibberellin-responsive element (GARE-motif), auxin-responsive element (TGA-element), ABA-responsive elements (ABRE and CE3), and ethylene-responsive elements (ERE) are present in the *ZmGRXCC* promoters, indicating that these *ZmGRXCC* genes could be involved in various phytohormone signaling pathways. Development- and metabolism-regulatory elements, such as Skn-1_motif, CCGTCC-box, CAT-box, and RY-element, are also present in the *ZmGRXCC* promoters. These results are consistent with previous reports showing that CC-type *GRXs* may participate in the control and regulation of organ development [20]. Furthermore, a large number of elements involved in various stress responses are also found in the *ZmGRXCC* promoters, such as stress-responsive elements (LTR and box S), MYB-binding site (MBS), dehydration-responsive element (DRE and T/G Box), evening element (EE), MYC-binding site (MYCR), and binding site of drought-inducible NAC TFs (NACR). These results indicate that the *ZmGRXCC* genes may participate in plant abiotic stress responses.

### 3.5. Expression Profile of ZmGRXCCs under Stress Treatments

**Figure 5:**
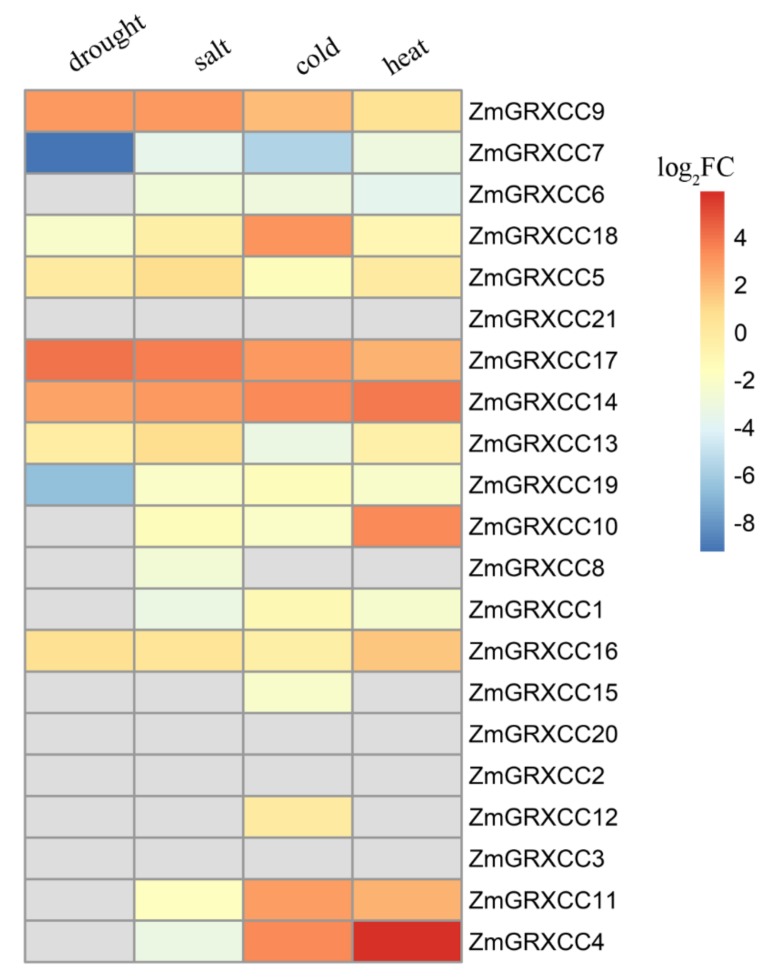
Transcriptomic analysis of *ZmGRXCC* genes. The log2-based fold change (FC) values were supplied to build the heatmap. The color scale ranged from blue, representing low expression, and passes through white and finally to red, representing high expression. The gray color shows missing data.

Studies have shown that the expression levels of CC-type *GRXs* are regulated by stress [12,13]. Based on the published RNA-seq transcriptome data (PRJNA244661 for drought and salt; PRJNA335771 for cold and heat), the log2 fold change relative to control were used to examine the gene expression profiles of the *ZmGRXCCs* in maize (Figure 5). In general, genes within the same subgroup (Figure 2A) showed similar expression patterns. *ZmGRXCC5*, *9*, *13*, *14*, *16*, *17,* and *18* were up-regulated above two-fold under drought, salinity, cold and heat, while others were down-regulated or undetected, indicated functional divergence of *ZmGRXCCs* in maize. Notably, *ZmGRXCC14* and *17* were up-regulated above two-fold by all the four treatments.

### 3.6. Natural Variations in ZmGRXCC14 Are Associated with Drought Tolerance in Maize

In order to further investigate whether the natural variations in any of the *ZmGRXCCs* are associated with the different drought tolerance levels of maize varieties, we conducted an association analysis for these genes. To assess potential associations between survival rates and *ZmGRXCCs*, we utilized previously reported methods and data [38,42]. Among the 21 identified *ZmGRXCC* genes, 12 were found to be polymorphic (Table 2), while the polymorphic information of the other nine genes was currently absent (minor allele frequency, MAF ≥ 0.05).

**Figure 6:**
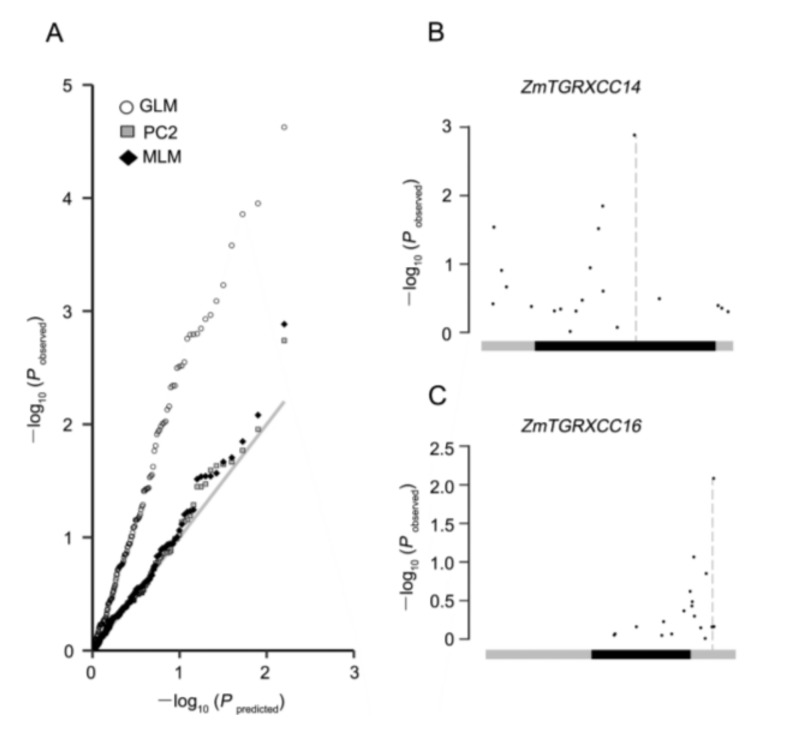
Association analysis of genetic variations in *ZmGRXCC14* and *ZmGRXCC16* with maize drought tolerance. (**A**), Quantile-quantile plots of estimated −log10(*P*) from *ZmGRXCC* gene family-based association analysis using three methods. The gray line is the expected line under the null distribution. The white square represents the observed *p* values using GLM; the gray square represents the GLM model with the first two principal components (PC2); the black diamond represents the observed *p* values using the MLM model incorporating both PC2 and a Kinship matrix. Schematic diagrams of *ZmGRXCC14* (**B**) and *ZmGRXCC16* (**C**), including protein coding regions (thick black line), are presented in the x-axis. The *p* value is shown on a −log10 scale.

Subsequently, three statistical models [42,43,44] were applied to identify significant genotypic and phenotypic associations (Figure 6A). The candidate gene association analysis detected significant associations between the genetic variations of *ZmGRXCC14* and *16* and drought tolerance under different models with a *p*-value ≤ 0.01 (Table 2; Figure 6B,C). However, under the standard mixed linear model (MLM), one significantly associated SNP contributing to the phenotype of drought tolerance was located at the CDS region of *ZmGRXCC14*, which suggests that this candidate gene is significantly associated with drought tolerance (*p*-value ≤ 0.005, −log_10_*P* = 2.86) (Figure 6B).

### 3.7. Expression Profile of ZmGRXCC14 and 17 Are Both Induced by Drought Stress

**Figure 7:**
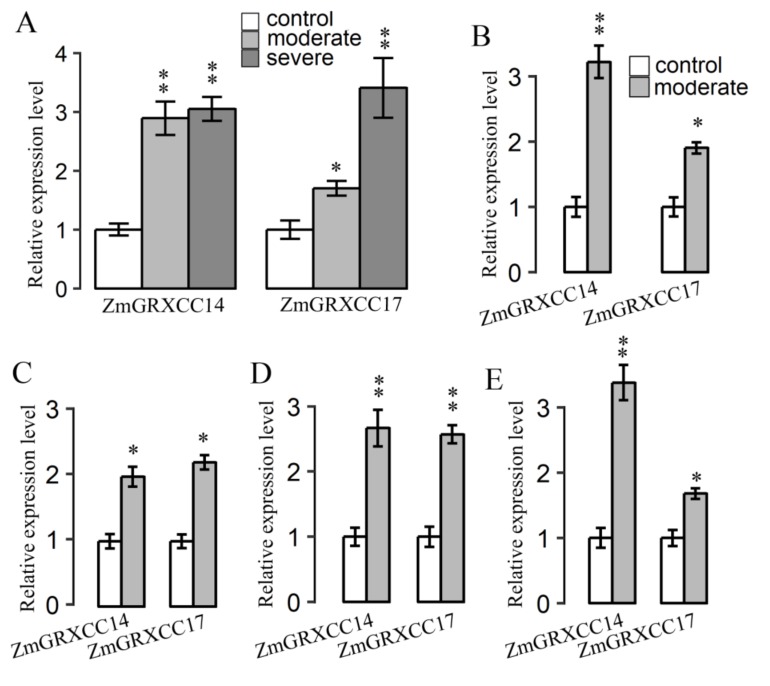
Expression patterns of *ZmGRXCC14* and *17* in seedlings (**A**), V12 (**B**), V14 (**C**), V16 (**D**), and R1 (**E**) under drought. *ZmUbi*-2 was used as an internal control for data analysis. The mean and SD were calculated from data of three biological replicates. *t*-test, * *p* ≤ 0.05, ** *p* ≤ 0.01.

*ZmGRXCC14* and *17* were consistently up-regulated by drought, salt, heat, and cold; we further analyzed their expression under drought treatment by RT-qPCR. Maize seedlings were treated with drought stress for 5 or 24 h and their RNAs analyzed to monitor their expression profiles. As shown in Figure 7A, *ZmGRXCC14* and *17* responded to drought stress in young maize seedling. Their expression levels were further assessed in adult leaves at four growth stages: V12, V14, V16, and R1. At the V8 stage, drought stress was applied by intercepting water, with the corresponding control being irrigated adequately (40% soil moisture). As shown in Figure 7, *ZmGRXCC14* and *17* were up-regulated by drought stress. These results showed that *ZmGRXCC14* and *17* may be involved in response to drought.

## 4. Discussion

Many reports indicate that CC-type GRXs are a class of small proteins that play an important role in plant development and abiotic stress responses [5,6]. However, there was no detailed analysis of the CC-type *GRX* gene family in maize, especially on their gene expression profile and genetic variation under drought stress. Therefore, we were prompted to conduct this study in an attempt to address this issue. Here, we identified a total of 21 maize CC-type *GRX* genes. Subsequently, we further systematically determined their phylogenetic relationship with rice, and *Arabidopsis* GRXs, pattern of drought-responsiveness, and association analysis of their natural variations with drought tolerance. Collectively, our data demonstrate that a few ZmGRXCC family proteins are likely to be involved in plant drought tolerance; in particular, *ZmGRXCC14* may function as an important gene for drought tolerance. The *ZmGRXCC14* gene will be of top priority for functional validation through overexpression and/or gene knockout in transgenic plants. Our results will not only facilitate the genetic improvement of drought resistance in maize but also increase our understanding of the biological functions of the CC-type *GRX* gene family.

At present, *GRX* family members described in various species are classified into five types, among which the CC-type are plant-specific [11,12,13]. In previous studies, there were 17 CC-type *GRX* genes in rice and 21 CC-type *GRX* genes in *Arabidopsis* [13]. Compared to rice or *Arabidopsis*, there is about one-fold duplication of CC-type *GRX* genes in maize. The unchanged ratio of gene numbers in species may suggest that the *ZmGRXCC* gene family has undergone a relatively conserved evolutionary history. On the other hand, as shown in our phylogenetic analysis (Figure 1), monocot GRX proteins cluster independently from *Arabidopsis* GRXs, suggesting a potential functional diversification between dicot and monocot *GRXs*.

In the present study, a total of 21 major regulatory elements are identified in the *ZmGRXCC* upstream regions, among which we find several types of motifs that may be responsive to drought stress and/or ABA hormone signaling (Figure 4). It is noteworthy that most of the *ZmGRXCC* gene promoters contain the ABRE motif. ABRE is a well-studied *cis*-element involved in ABA-induced gene expression, and it has been found to require a coupling element (CE) to achieve ABA induction [45,46]. Interestingly, we find that 12 *ZmGRXCC* promoters contain both ABRE and its CE3, suggesting that these genes may function in the ABA-mediated drought signaling pathway [12]. More importantly, 17 of the 21 *ZmGRXCC* promoters contain the JA-responsive element, implying that these genes may also be involved in the jasmonic acid signaling pathway [11,18]. Taken together, the mechanism by which *ZmGRXCC* genes respond to drought stress is likely through the ABA- and/or JA-mediated pathway.

To our knowledge, although the relationship between CC-type GRX proteins and plant stress was studied [21,23,47], the dynamic drought-responsive expression patterns of *ZmGRXCC* genes had not been reported. Analysis of the expression patterns of *ZmGRXCC* genes can give clues to their possible functions, and pave the way for future research. In general, different members of *ZmGRXCCs* respond differently to drought, salinity, cold, and heat, suggesting that they not only carry different functions between subgroups but also among members of the same subgroup (Figure 5). Seven of the twenty-one *ZmGRXCCs*, including *ZmGRXCC14* and *17*, are up-regulated by at least one of the drought, salinity, cold, and heat treatments, while others are not (Figure 3). Notably, *ZmGRXCC14* and *17* are further validated to be up-regulated at seedlings and adult leaves by qPCR analysis (Figure 7). Importantly, the rice orthologs of *ZmGRXCC14* and *ZmGRXCC17* are also induced by plant hormones and abiotic stress [13], indicating that these genes may function as key mediators of drought tolerance in maize.

Identification of the key genetic components underlying drought is critical and will serve as the foundation for crop genetic improvement. Among the 21 *ZmGRXCCs* analyzed in this paper, the genetic variations of *ZmGRXCC14* and *ZmGRXCC16* are significantly associated with drought tolerance (*p*-value ≤ 0.01, MLM) (Table 2). While *ZmGRXCC14* is the most significantly (*p*-value ≤ 0.005, MLM) associated with drought tolerance among the *ZmGRXCC* gene family, the natural variation in the *ZmGRXCC14* CDS may contribute to maize drought tolerance (Figure 6). In comparison with *ZmGRXCC16*, *ZmGRXCC14* is detected at a higher level under various stress (Figure 5). More notably, *ZmGRXCC14* is significantly induced by drought stress at both young seedlings and adult leaves (Figure 7). These results indicate that *ZmGRXCC14* is involved in plant drought response. In summary, we suggest that *ZmGRXCC14* may be an important positive regulator of drought tolerance through analyses of gene expression and natural variations.

## 5. Conclusions

In this report, we identified 21 maize CC-type GRX genes in the maize genome. Phylogenetic analyses revealed that the *ZmGRXCC* gene family had a conserved evolutionary history. Protein domain analysis indicates that most of the 21 ZmGRXCCs share similar structures to their homologs. Promoter analysis show that *ZmGRXCCs* have a diverse profile of *cis*-elements associated with phytohormones, growth, development, and abiotic stress. Analysis of their differential expression profiles upon stress at various developmental stages, including seedlings and adult leaves, as well as the expression profiles in 15 tissues, suggest a functional divergence of ZmGRXCC genes. Importantly, *ZmGRXCC14* is up-regulated in both seedlings and adult leaves, and the natural variations in *ZmGRXCC14* are significantly associated with drought-stress tolerance, implying *ZmGRXCC14* is an important candidate gene for maize drought tolerance improvement. The findings presented here will enhance understanding of the role of *ZmGRXCCs* under drought.

## Figures and Tables

**Table 1:** Detailed information for the 21 *ZmGRXCC* genes identified in the *Zea mays* genome.

Identifier	Gene Symbol	Chromosome	Class	Redox Site	ALWL-Motif
GRMZM2G413315	*ZmGRXCC1*	1	CC	CCMC	ALWV
GRMZM2G469994	*ZmGRXCC2*	1	CC	CCMC	ALWV
GRMZM2G480903	*ZmGRXCC3*	2	CC	CCMC	ALWL
GRMZM5G892308	*ZmGRXCC4*	2	CC	CCMC	ALWL
GRMZM2G303044	*ZmGRXCC5*	3	CC	CCMA	ALCA
GRMZM2G110286	*ZmGRXCC6*	3	CC	CCMC	ALWV
GRMZM2G052796	*ZmGRXCC7*	3	CC	CCMC	ALWV
GRMZM2G403680	*ZmGRXCC8*	3	CC	CCMC	ALWL
GRMZM2G023237	*ZmGRXCC9*	3	CC	CCLS	ALWL
GRMZM2G371063	*ZmGRXCC10*	4	CC	CSMG	ALWL
GRMZM5G860607	*ZmGRXCC11*	4	CC	CSMG	ALWL
GRMZM2G470756	*ZmGRXCC12*	5	CC	CCMC	ALWL
GRMZM2G318213	*ZmGRXCC13*	6	CC	CCMC	ALCG
GRMZM2G318180	*ZmGRXCC14*	6	CC	CCLS	ALWL
GRMZM2G442791	*ZmGRXCC15*	7	CC	CCMC	ALWL
GRMZM2G441906	*ZmGRXCC16*	8	CC	CCMA	ALCA
GRMZM2G311898	*ZmGRXCC17*	8	CC	CCLS	ALWL
GRMZM2G178886	*ZmGRXCC18*	8	CC	CCLS	ALWL
GRMZM2G337706	*ZmGRXCC19*	10	CC	CPMC	AIWL
GRMZM2G457898	*ZmGRXCC20*	10	CC	CCMC	ALWV
GRMZM2G303536	*ZmGRXCC21*	10	CC	CPMC	DIWL

**Table 2:** Association analysis of natural variation in *ZmGRXCC* genes with respect to drought tolerance at the seedling stage in the maize diversity panel. GLM, PC2, and MLM stand for general linear model, general linear model with the first two principal components, and mixed linear model, respectively.

Locus ID	Gene Name	Polymorphic	GLM	PC2	MLM	
			*p* ≤ 0.01	*p* ≤ 0.01	*p* ≤ 0.01	*p* ≤ 0.005
GRMZM2G413315	*ZmGRXCC1*	1	-	-	-	-
GRMZM2G469994	*ZmGRXCC2*	-	-	-	-	-
GRMZM2G480903	*ZmGRXCC3*	1	1	-	-	-
GRMZM5G892308	*ZmGRXCC4*	-	-	-	-	-
GRMZM2G303044	*ZmGRXCC5*	43	-	-	-	-
GRMZM2G110286	*ZmGRXCC6*	1	-	-	-	-
GRMZM2G052796	*ZmGRXCC7*	3	-	-	-	-
GRMZM2G403680	*ZmGRXCC8*	-	-	-	-	-
GRMZM2G023237	*ZmGRXCC9*	23	-	-	-	-
GRMZM2G371063	*ZmGRXCC10*	-	-	-	-	-
GRMZM5G860607	*ZmGRXCC11*	-	-	-	-	-
GRMZM2G470756	*ZmGRXCC12*	1	-	-	-	-
GRMZM2G318213	*ZmGRXCC13*	11	-	-	-	-
GRMZM2G318180	*ZmGRXCC14*	20	10	1	1	1
GRMZM2G442791	*ZmGRXCC15*	-	-	-	-	-
GRMZM2G441906	*ZmGRXCC16*	20	4	-	1	-
GRMZM2G311898	*ZmGRXCC17*	1	-	-	-	-
GRMZM2G178886	*ZmGRXCC18*	34	10	-	-	-
GRMZM2G337706	*ZmGRXCC19*	-	-	-	-	-
GRMZM2G457898	*ZmGRXCC20*	-	-	-	-	-
GRMZM2G303536	*ZmGRXCC21*	-	-	-	-	-

## Supplementary Materials

Supplementary Materials can be found online at https://www.mdpi.com/2073-4425/10/8/610/s1. Table S1. Sequence alignment used to generate figure 1, Table S2. PCR Primer Sequences Used for This Research.
